# Bioherbicidal Potential of the Essential Oils from Mediterranean Lamiaceae for Weed Control in Organic Farming

**DOI:** 10.3390/plants10040818

**Published:** 2021-04-20

**Authors:** Giuseppe De Mastro, Jihane El Mahdi, Claudia Ruta

**Affiliations:** 1 Department of Agricultural and Environmental Science, Università degli Studi di Bari Aldo Moro, Via Amendola 165/a, 70125 Bari, Italy; claudia.ruta@uniba.it; 2 CIHEAM Centre International des Hautes Études en Agriculture Méditerranéenne, IAM Mediterranean Agronomic Institute of Bari, Via Ceglie 9, 70010 Valenzano, Italy; elmahdijihane@gmail.com

**Keywords:** terpenes, mechanism of action, germination inhibitors, crops

## Abstract

In all farming systems, weeds are the most expensive pest to manage, accounting for 30% of potential losses. In organic farming, the problem may be further amplified by restrictions on herbicides, thus making weeds the main problem faced by organic farmers in the field. In this sense, much research is focusing on the allelopathic potential of plants as an ecological weed control tool. Many plant species can release allelopathic compounds with high phytotoxicity that can be used in weed control. Species belonging to the Lamiaceae family have been studied widely for this purpose, and their essential oils (EOs) appear to be promising bioherbicides. However, there are still many challenges for their development. Considering these aspects, a review of the bioherbicidal effect of EOs from Mediterranean Lamiaceae could help identify the most effective ones and the challenges for their actual development.

## 1. Introduction

The emerging worldwide need to find alternatives to synthetic herbicides for sustainable weed control has prompted considerable interest in exploiting the natural herbicidal potential in plants [1]. Bioherbicide sources are sought out by both conventional and organic farming systems: the former wish to identify new sites of action to cope with weed resistance, the latter seek potent alternatives to synthetic herbicides that can be integrated in an overall management approach [2]. In this context, weed control research has recently focused on extracts from allelopathic species. These are species that can release secondary metabolites able to interfere with the growth and functions of surrounding plants [3].

A well-established group of allelopathic plants is that of the Lamiaceae family. They are known to contain high concentrations of volatile allelochemicals, which are responsible for their aroma, and are reported to give the species a competitive advantage in their natural habitats [4]. In this context, extracts of different Lamiaceae species were studied extensively and found to inhibit the germination and growth of many weed species [5,6,7,8,9,10,11,12]. Essential oils (EOs) from species such as oregano, thyme, rosemary, sage and mint are reported to be particularly strong bioherbicide candidates.

The phytotoxic effect of these species extracts, notably EOs, has mainly been linked to the presence of volatile bio-active compounds such as α-pinene, limonene, 1,8-cineole, carvacrol, camphor and thymol, which have been shown to have varying individual phytotoxicity levels [4,11,12,13,14,15]. Some phenolic compounds present in the EOs were also reported to be involved in allelopathic interactions and were even used to develop commercial bioherbicides. The mechanisms by which these allelochemicals can affect weeds was not discussed in detail. Only a few individual compounds were studied [16,17,18], in addition to the mechanism behind some naturally occurring allelopathic interactions [16,19,20,21].

Although there are numerous studies reporting on the successful use of EOs in weed control, to date there are still many constraints limiting their practical application in commercial bioherbicides. For instance, the role of the EO composition is still not clearly described. The mechanisms of action and the observed selectivity are also very poorly understood, limiting their rational implementation. Moreover, studies concerning the possible side effects of these EOs on beneficial soil microorganisms are still lacking.

This review will address all the above-mentioned issues pertaining to the use of EOs from Lamiaceae species in weed control in order to further highlight their potential uses and perspectives for future studies. It will also review the literature on certain species most frequently studied.

## 2. Weed Management in Organic Agriculture

### 2.1. Objectives and Methods of Weed Management in Organic Agriculture

To understand the aims of weed control in organic farming one must understand the overall objective of this production system. How to maximize yields and economic gain are major concerns for organic farmers, like others. However, in this system the emphasis is on the long-term outcome and overall health of the soil, plants, animals, and humans rather than just immediate maximum profitability [22]. In this context, many operational techniques have been defined to meet what could be regarded as the main goals of organic farming. Kirschenmann [23] presented four techniques related to the different aspects of management: nutrients, insects, plant disease and weeds. As for the latter, the overall goal was “to achieve weed control using crop rotation systems to deprive weeds of favorable growing conditions”. Liebman et al. [24] defined more detailed, equally important objectives. These can be summarized as follows:

- reducing weed density to a tolerable level, instead of targeting 100% control or total suppression;
- reducing the damage that a given density of weeds can cause, by increasing the competitive ability of crops and minimizing that of weeds through different preventive and cultural tools (competitive varieties, fertilization, irrigation and false seed beds);
- shifting the composition of weed communities to less aggressive, more easily managed species.

These goals may be achieved through a knowledge-intensive process. A good understanding of weed ecology, of the site and of the crop-weed interactions is required.

Kirschenmann [23] claims in this context that the organic system seeks to farm like nature, which implies knowing and understanding the natural processes and incorporating those principles on the farm.

Managing weeds in an organic system is more complicated than in a conventional system, mainly because of restrictions on the use of herbicides [25]. The latter are easy to apply and aggressively marketed, although in recent years there has been a tendency to restrict the use of chemicals in agriculture to preserve human health and the environment. [24]. Therefore, a combination of tools and practices that take into account the natural system’s cycles and interactions are increasingly being adopted to manage weeds. The management system is consequently an integrated approach, one that adopts different preventive, cultural, and direct control methods to achieve the goals detailed in the paragraph above [25,26].

- Preventive methods

Prevention aims at reducing the density of the actual weed vegetation by exhausting the potential weed vegetation (e.g., weed seedbank in the soil). This means reducing in-crop weed emergence and weed seed dispersal. Operational techniques include crop rotation, tillage systems, the false seedbed technique, cover crops, mulching and soil solarization.

- Cultural methods

Cultural methods are commonly used to reduce the need for direct weed control (e.g., herbicides) and increase its effectiveness. This is achieved by choosing cultural techniques that favor the competitive ability of crops against weeds. Cultural weed management techniques include crop genotype choice, planting pattern, polyculture production systems, fertilization, and irrigation strategies.

- Direct methods

Direct methods aim at intervening directly during the crop cycle to eliminate the weeds, mainly using physical or chemical tools.

Physical tools include mechanical weeding or cultivation, which is based on a variety of equipment. Recent technical innovations focusing on intra-row weed control in arable and vegetable crops have proven to be effective [26]. Robotic control is another technological innovation increasingly adopted by organic farmers. Post-emergence flame weeding, which can be used after planting or crop emergence, is another physical technique. Flaming can be used to eliminate weeds within the row where cultivation is difficult or can considerably damage the crop. Lastly, manual weeding is also widely used in organic management, notably when other measures are not feasible, such as within rows or when the crop is susceptible to damage by cultivation.

Bioherbicides are the main chemical tools. The latter are compounds and secondary metabolites derived from microbes, phytotoxic plant extracts or single compounds [2].

### 2.2. Challenges of the Current Weed Management Methods in Organic Agriculture

Effective weed management in the organic system necessarily involves integration of the highest number of available tools and approaches [27,28]. Preventive methods based on ecological principles and building biodiversity such as rotation or cover cropping are of particular interest to this system. However, direct methods are still contributing the most to weed control in many organic farms [27,29,30]. Therefore, serious weed competition problems may arise when few direct control methods are available or applicable. Moreover, those currently available present serious limitations (Table 1).

By studying existing limitations in direct weed control methods, it is possible to define research needs and opportunities. This is particularly true of bioherbicides, especially considering the limitations and the numerous potential sources of active compounds in nature. This research field is increasingly important due to increasing consumer awareness and environmental problems related to synthetic herbicides (residues, weed resistance). Usually, the EOs have various modes of action, and therefore it is more complicated for weeds to develop easily resistance against them [10,31]. This aspect increased the attention to their bioherbicidal potential, widely investigated in the hope of finding effective, viable products that can meet registration requirements.

## 3. The Use of Plant-Based Bioherbicides

### 3.1. Bioherbicidal Potential in Plants

The interest in exploiting the natural herbicidal potential of plants stems from a worldwide need to find new sustainable weed control strategies [1].

**Figure 1:**
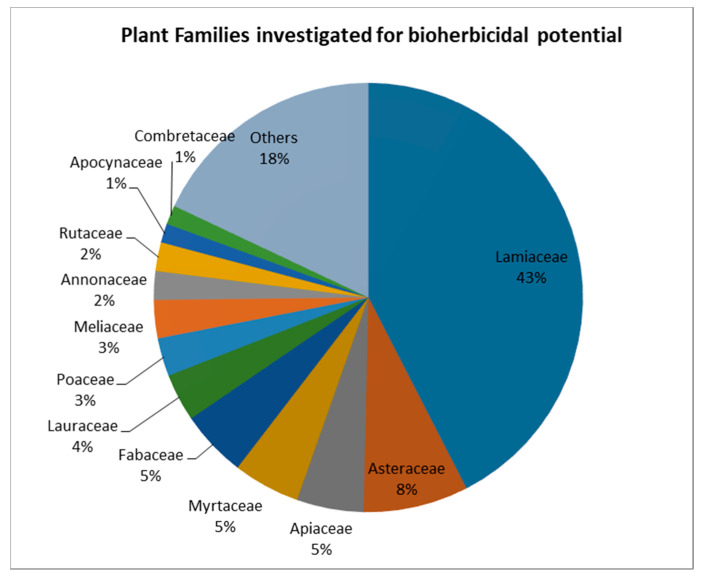
Plant families studied for their bioherbicidal potential. (Source: elaborated from a search on Scopus, 2019).

As plants are the richest source of active organic compounds on Earth, the bioherbicidal potential of a long list of plant species has been explored [32]. A Scopus literature search using the keywords « Bioherbicides AND plant extracts » and « Bioherbicides AND Essential oils » found 130 articles (excluding review articles) on the bioherbicidal potential of species from 38 different families (Figure 1).

Plant species can be considered for investigations due to their known composition in terms of biologically active compounds, or an observed allelopathic effect in their natural environment. Allelopathy is a characteristic of many plant species, and can be defined as a form of interaction between plants through chemical inhibitors released from living or decaying tissues [3]. Evidence that some plants are able to inhibit the growth of other plants in their surroundings has long been known and reported, and studies have linked these interactions to the presence of compounds named “allelochemicals” [4,21,33]. This can justify the high interest in the Lamiaceae family species—accounting for 43% of the total studied species from the papers covered by the search—which are known to possess high concentrations of volatile allelochemicals. In this context, much effort has been made to extract allelochemicals from plants and test their bioherbicidal activity in bioassays; many were successful (Table 2). Other studies evaluated the activity of single compounds isolated from plants, such as flavonoids, alkaloids and terpenoids. These different classes of secondary metabolites have different importance in term of bioherbicidal activity. In extracts from the same species, they can occur in different proportions, depending on the type of extract and the extraction method [34].

### 3.2. Types of Active Compounds and Plant Extracts Tested as Bioherbicides

#### 3.2.1. Active Compounds with Bioherbicidal Potential

The term active compounds usually refers to secondary metabolites occurring in plants, known for having diverse biological activities. These are the compounds with no relevance to vital functions (like respiration, photosynthesis and reproduction), but involved in interactions between plants and their surrounding environment, notably as part of their mechanism of defense against stress [40,41]. Secondary metabolites in plants have been classified differently by different authors; a recent review by Yasri et al., [42] defined four main groups: terpenoids, phenolics, sulphur-containing secondary metabolites and nitrogen-containing secondary metabolites. Not all of these groups of secondary metabolites were found to be implicated in allelopathic interactions or showed a bioherbicidal potential. Although some authors included amino acids and proteins among the phytotoxic compounds, terpenoids and phenolics were the ones most frequently studied [4]. Only these two groups will therefore be considered in detail in this paragraph.

(a)—Terpenoids

The terpenoid group is present in the majority of secondary metabolite classifications and is reported to be very important in allelopathic interactions [4,21,43]. The compounds of this group, sometimes referred to as volatile allelochemicals, can be divided into monoterpenes, sesquiterpenes, diterpene, triterpenes and polyterpenes [42]. Monoterpenes are the major constituents of essential oils and have been shown to inhibit seed germination and seedling growth [14]. They are the most frequently described secondary metabolites for bioherbicidal activity [4,14,42,43,44,45,46]. Some monoterpene-based commercial herbicides have been developed such as cinmethylin, which is a derivative of 1,4-cineole [14]. Compounds having different chemical functions (Table 3) belonging to this sub-group were found to have varied inhibition effects.

Most authors considered that ketone-containing compounds such as camphor and pulegone are the most toxic, followed by alcohol compounds such as cineol and citronellol, and by ether, diene and monoene compounds such as α-pinene, which are the least toxic [47]. This was confirmed by many other authors [14,47]. Considering that plant species and chemotypes have different monoterpene composition, the phytotoxicity of extracts can vary between plant materials with different percentages of effective compounds (e.g camphor). However, there is no clear evidence reported in the literature as to how the active compounds of a plant extract define its activity level. In other words, it is not clear whether the observed toxic effect of plant extracts is due to the potent phytotoxicity of a single compound or to the synergic action of many constituents.

(b)—Phenolics

**Figure 2:**
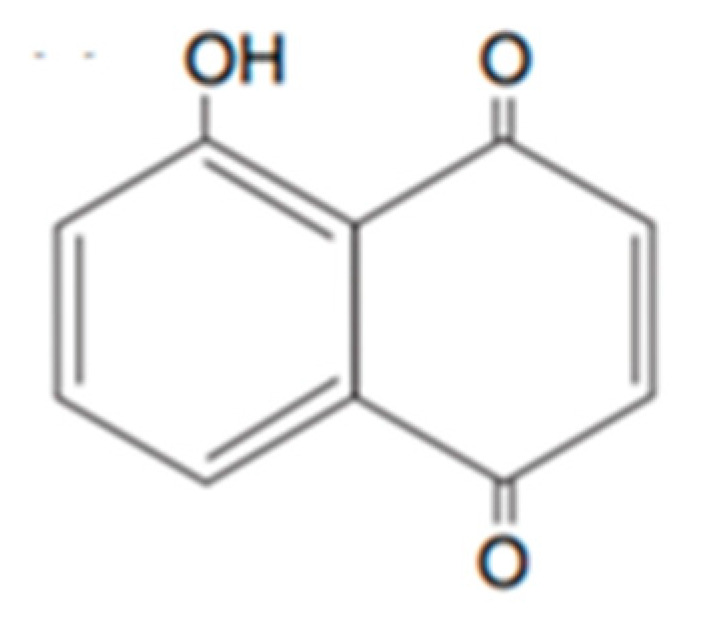
Representative structure of juglone.

Plant phenolics include phenolic acids, flavonoids and tannins. They are synthetized by plants as a response to ecological and physiological conditions, mainly when they are under biotic or abiotic stress [54]. Like the terpenoids, an important focus exists on the identification of phenols with bioherbicidal activity. This was attributed to the easiness of their extraction and their water solubility [47]. They are usually the main components in aqueous and organic solvents extracts, and their polarity determines the type and amount of phenols extracted. An example of a well-studied phenolic for this effect is juglone (Figure 2) produced by walnuts [55].

#### 3.2.2. Types of Plant Extracts Tested for their Bioherbicidal Activity

The extraction method is a determining factor in the recovery of active compounds from plants, considering that secondary metabolites of different groups have varying chemical properties (volatility, polarity etc.). For instance, anthocyanins, tannins, saponins and terpenoids can be recovered using water, whereas polyphenols, flavonoids, flavones and alkaloids require organic solvents [34]. Like the terpenoids, much research focuses on identifying phenols with bioherbicidal potential. This is because they are easily extracted and are soluble in water [43]. They are usually the main components in aqueous and organic solvent extracts, the polarity of which determines the type and amount of phenols extracted. An example of a well-studied phenolic with this effect is juglone (Figure 2), produced by walnuts [55]. These different extracts often show different levels of toxicity. In a study conducted to test *Calamintha nepeta* L. (Savi) as a source of phytotoxic compounds, solvents of varying polarity (*n*-hexane, chloroform, ethyl acetate and n-butanol) were used to fractionate the leaves’ methanol extract. The study defined the following hierarchical phytotoxicity: ethyl acetate > *n*-hexane > chloroform > *n*-butanol [56]. 

In general, three main groups of extracts can be found in the literature: essential oils (EOs), aqueous extract and organic solvent extracts.

- Essential oils: Sometimes called volatile oils, these are natural substances that can be extracted from aromatic plants by distillation or by appropriate mechanical process without heating. EOs mainly contain compounds that can be volatilized during extraction, making this an effective means of extracting plant terpenoids in the purest form [18,57]. These, the most frequently tested extracts from aromatic plants, can cause higher phytoxicity compared to aqueous or organic solvent extracts [38,56,58].
- Aqueous extracts: These are obtained simply by soaking in water ground dry material from plants, from which water-soluble compounds are extracted. Several phenols are water soluble and can successfully be extracted using this method. Aqueous extracts have been used to investigate the bioherbicidal potential of many plants and have been found to produce significant effects mainly at the highest tested concentrations [1,59,60].
- Organic solvent extracts. This group consists mainly of phenols. As the type of solvent (mainly differing in polarity) affects the amount and type of phenols extracted, authors have used various solvents. Methanol, ethanol, acetone, ethyl acetate, n-hexane and chloroform are among those used most frequently [54]. The choice of solvent depends on the types of phenol present in the tested plant, and many authors have tested different ones simultaneously in order to compare the composition and phytotoxicity of the resulting extract [56,61].

### 3.3. Modes of Action of Plant Allelochemicals

After investigating the type of active compounds in the plants’ extracts, research has also explored the mechanisms of toxicity to weeds. The most frequently described effects are from single allelochemicals rather than whole plant extracts; the modes of action of terpenoids and phenolic acids, which are reported to be the most relevant secondary metabolites in allelopathic interactions, have been studied by many authors. However, studies on this topic are still lacking and the mechanism of only a few phytotoxic compounds has been described. This paragraph therefore focuses mainly on toxicity mechanisms reported for single allelochemicals, as well as allelopathic mechanisms observed in nature. Note that to determine the stage in which plants are most sensitive to allelochemicals, the latter were often tested in two different periods (pre-germination and post-emergence), and different features and mechanisms were analyzed accordingly.

#### 3.3.1. Effect on Cells Division, Elongation and Structure

The size and weight of weed seedlings are the features most often measured to assess their reaction to the application of allelopathic compounds. The application of plant extracts usually results in a significant decrease of these parameters compared to the control. The substances undoubtedly affect the responsible physiological processes: cell division and elongation [21]. In this sense some studies have reported that some allelochemicals affect mitosis: the process was either slowed down [19], interrupted in the anaphases or hindered altogether [16,19,20,62,63]. All the cited studies measured the number of cells and their ultrastructure at specific times as indicators. Muller [16] also reported that volatile terpenes extracted from *Salvia leucophylla* Greene (mainly cineol and camphor), prevented the elongation of root and hypocotyl cells. Cineole is in fact the most widely described of all monoterpenes [64]. It is generally reported to strongly inhibit all stages of mitosis. The suggested mechanism can therefore result in considerable damage to weeds by reducing their growth or retarding it, which can give the crop a competitive advantage.

#### 3.3.2. Effect on the Cells Membrane Integrity and Permeability

Cell membrane integrity is critical for cell functions and survival. Any alteration may compromise its role as a barrier, affecting permeability to nutrients or toxins or inducing the leakage of solutes [65,66]. A number of allelochemicals seem to alter plant cell membranes. Due to lipophilic nature of the cell membranes, monoterpenes can cause their destruction by increasing permeability or inhibiting enzymes [18]. Moreover, some monoterpenes are reported to induce oxidative stress; α-pinene, for example, caused lipid peroxidation when applied to young seedlings of *Cassia occidentalis* L., resulting in an increase in solute leakage [48]. Furthermore, some compounds produced changes to the permeability of membranes; Varona et al. [67] found that linalool caused an increase in permeability, whereas Muller et al. [16] found that permeability decreased after applying cineole and dipentene from *S. leucophylla*. This suggests that allelochemicals can result in important damage to weeds by acting at the membrane level.

#### 3.3.3. Effect on Photosynthesis

There is evidence of a relationship between the visible effects on weeds and photosynthetic functions. Early studies found a correlation between the reduction in growth caused by a plant-extracted phenolic substance, “scopoletin”, and net photosynthesis in *Amarantus retroflexus* L. [68]. Many other studies found that a number of phenolic acids affect photosynthesis, and this was linked to changes to stomatal conductance or to plant chlorophyll contents [68,69]. Furthermore, many monoterpenes were also found to inhibit photosynthesis and chlorophyll synthesis [70]. Citronellol and 1,8-cineole, for example, showed a similar effect on the invasive weed species *Ageratum conyzoides* L.: its chlorophyll content decreased by 60% and 66%, respectively [18,50]. Eugenol, another monoterpene, has a similar effect: it induced photosynthetic inhibition by reducing chlorophyll content in *C. occidentalis* and *Bidens pilosa* L. [71]. These examples suggest that photosynthesis-related processes could be behind the observed damage. However, only a few of the allelochemicals were tested, and the actual cause-effect between the described processes is not yet well understood. 

#### 3.3.4. Effect on Nutrients Availability and Uptake

Because of the observed effects on the root appearance, some research has focused on whether allelochemicals inhibit nutrient uptake [21]. The uptake of phosphorous, potassium, calcium and zinc, for example, was affected either by the direct application of some phenolic acids or by growing plants in association with allelopathic species [72,73,74,75,76,77]. Moreover, some early studies found that toxic excretions from plants reduce the availability of nutrients by affecting nutrient cycling mechanisms; mineralization, for example, was suppressed by the root excretion of some natural forest vegetation due to its toxicity to the nitrification process [78]. This suggests that phytotoxic compounds from plants may affect soil microbial activity, which plays an essential role in making important nutrients like nitrogen available to plants.

All the presented modes of action suggest that allelochemicals have a strong potential as weed control tools. However, they also highlight the many challenges to their practical application. For instance, no clear selectivity can be concluded from the reported mechanisms, which means that crops may also be susceptible. Moreover, the impact on crop and soil health is also of concern if the allelochemicals have a detrimental effect on beneficial soil microbes. This, in addition to other possible challenges, will be detailed in the next paragraph.

### 3.4. Challenges and Perspectives to the Use of Plant Extracts as Bioherbicides

#### 3.4.1. Challenges to the Use of Plant Extracts as Bioherbicides

(a)—Unclear selectivity

Although allelochemicals may affect specific functions like photosynthesis or respiration, they lack site specificity, which excludes their use as selective bioherbicides. This also means they could be phytotoxic to crops and must be managed carefully when applied. However, many studies that tested plant extracts on different weed species revealed varying degrees of sensitivity. In most cases monocots were more resistant than dicots [7,8,79]. Moreover, it was frequently reported that many crops were less affected than weeds; for instance, when applying the EO of *Satureja hortensis* L. and *Laurus nobilis* L. at low concentrations, *A. retroflexus* germination decreased significantly whereas tomatoes were unaffected. However, at the highest tested concentration, tomato germination also decreased, albeit at a lower rate than *A. retroflexus* [8]. Similar results were obtained when applying *Origanum onites* L. and *Rosmarinus officinalis* L. on *Avena sterilis* L., *Sinapis arvensis* L. and a number of wheat cultivars, where the latter were less affected [7]. This suggests that careful dosage may resolve phytotoxicity to crops. Nevertheless, studies were not able to explain this variation in sensitivity, which makes it difficult to predict and exploit. Further research is required to better understand the mechanism of action of different allelochemicals and the synergies by which they operate in plant extracts.

(b)—Toxicity to soil microorganisms

Organic farming relies on soil health and natural soil processes to satisfy crop nutrient needs and ensure long term fertility. In fact, one of the serious drawbacks of synthetic chemicals is their impact on soil biodiversity and their harmful effect on beneficial organisms. Plant extracts with similar effects cannot be recommended regardless of their possible effectiveness on weeds. Only a few studies have addressed this important aspect. As mentioned in the “effect on nutrients uptake and availability” paragraph, some allelochemicals may be detrimental to nitrification bacteria [78]. Moreover, many plant extracts, notably those from the Lamiaceae family, have been shown to possess antimicrobial properties [80,81,82]. Doubts may thus arise about their possible harmful effects on soil microbes. However, other studies have reported a positive impact in this respect; volatile substances from alfalfa (*Medicago sativa* L.), for example, induced a rapid increase in microbial respiration and fungi mycelium growth when added to the soil. Results thus suggest a possible beneficial effect on the initial colonization stage of plant residue decomposers [83]. The different findings may be ascribed to variations in the concentration of compounds in contact with microorganisms.

In summary, the soil microbial community seems to be affected by allelochemicals (either negatively or positively). Hence, when assessing the use of plant extracts as agrochemicals, care should also be taken to detect any possible negative repercussions on soil life, a crucial component of any sustainable management strategy.

(c)—Degradation of plant extracts in the environment

While the incorporation of allelopathic plant species biomass into the soils is constrained by the difficulty in accumulating active concentrations [21], the direct use of concentrated extracts is mainly limited by susceptibility to environmental elements. Once released in the environment, the extracts are subject to decomposition either by microorganisms or by chemical reactions. Blum [84] reported in the book chapter «Fate of phenolic allelochemicals in soils − the role of soil and rhizosphere microorganisms» that because microorganisms use phenolic acids as a source of carbon or energy, they are thus more subject to microbial transformation and utilization than to other processes (ionization, oxidation, sorption onto soil particles, fixation into the recalcitrant organic matter (e.g., polymerization)). In this chapter the author reports results from many studies suggesting that this degradation is very likely and that phenolic acids are unlikely to produce any phytotoxic effects. Moreover, Marmulla and Harder [85] report that monoterpenes such as d-limonene, α-pinene, γ-terpinene and terpinolene are readily biodegradable. They also found that different monoterpenes show different susceptibility to degradation. In addition, many allelochemicals are highly susceptible to spontaneous decomposition; abiotic photochemical processes in the atmosphere can result in lifetimes of minutes to hours, as cited by the same authors [21]. However, very little is known about their abiotic degradation in soil [81]. These aspects suggest that allelochemicals may lack the necessary persistence to be effective bioherbicides. This may be remedied by selecting critical stages of weed growth. Even a brief period of phytotoxicity could affect the competitive ability of weeds with respect to crops [81]. Another approach recently under study is the use of innovative formulations that could regulate the rate of release without compromising the desired concentration levels. For instance, experiments with rosemary EO encapsulated in a starch matrix were successful [86].

#### 3.4.2. Perspectives for the Use of Plant Extracts as Bioherbicides

Despite the many constraints, the use of plant extracts for weed control is still considered a field with great potential. However, to address limitations, research should focus on better understanding the phenomena in terms of:

- Linking the observed effects of extracts to the action of specific compounds and their synergies;
- Defining the mechanisms behind the phytotoxicity to enhance it and understand the selectivity;
- Defining the most sensitive stages of weed development to increase effectiveness and tackle the problem of the limited duration of the effect;
- Defining innovative formulations that take into consideration the interactions of the extracts with field conditions (soil texture, microorganisms and abiotic factors such as light and temperature);
- Defining innovative techniques for the cultivation and extraction of essential oils to guarantee the commercial feasibility of a mass production large quantity of EOs;
- Defining formulations that allow for containing the concentrations of EOs within technical limits for an easy application on an agricultural scale.

## 4. Examples of Lamiaceae Species with Bioherbicidal Potential

### 4.1. Oregano

In the literature, oregano is used to refer to a number of species in different genera and families, the leaves and flowers of which have a common characteristic odor and flavor [87,88]. The major oregano species belonging to the Lamiaceae family, *Poliomintha longiflora* L., *Origanum vulgare* L. and *O. onites*, are mainly found in the Mediterranean basin [87,88]. *O. vulgare*, and *O. vulgare* subsp. *hirtum* (Link) Iestwart plants in particular are extremely rich in essential oils (up to 8% dry weight) [87]. The famous odor and flavor of these species are mainly linked to their carvacrol content, which in addition to thymol, p-cymene and γ-terpinene, is known to be the major component of oregano essential oil [31,89,90]. As for the bioherbicidal effect, pure carvacrol and the EO from some species (mainly *O. vulgare* and *O. onites*) have been widely investigated, with interesting findings. In a study by De Mastro et al. [31] on *A. retroflexus* and *Lolium perenne* L., carvacrol at the concentration of 0.3 µL/mL completely inhibited the germination of both species. The same study assessed the application of dry biomass of an oregano hybrid (*O. vulgare* ssp. *virilidum* x *O. vulgare* L. ssp. *hirtum*) in a pot trial, and promising results were obtained using 20 g per kg of soil. In another investigation by Atak et al. [7], *O. onites* EO was tested against *A. sterilis* and *S. arvensis* L., and severe inhibition was observed on both species starting from 0.2 µL/mL. In this experiment the EO was also tested on a number of wheat cultivars, which were found to be less sensitive: this led the authors to suggest a possible dosed application of the EO as a bioherbicide in wheat fields. Ibáñez and Blázquez [91] also tested an EO dominated by carvacrol (60.42%) against *Portulaca oleracea* L., *Lolium multiflorum* L. and *Echinochloa crus-galli* L. They found that germination was completely inhibited in all the species starting from the lowest tested dose: 0.125 µL/mL. Hanana et al. [92] found the same high effectiveness at low doses on some important weed species (*S. arvensis* L., *Phalaris paradoxa* L. and *Lolium rigidum* Gaud) using a carvacrol- and δ-terpinene- rich EO from *O. vulgare*. Its high yield and strong anti-germination and phytotoxic effect make oregano EO a promising bioherbicide candidate.

### 4.2. Rosemary

Rosemary (*R. officinalis*), an evergreen shrub that can grow up to 2 m high, has aromatic leaves and flowers rich in essential oils [87,93]. Native to the Mediterranean region, it is characterized by high tolerance to heat, drought, and poor, dry, sandy and rocky soil types [87]. It is grown in different parts of the world, such as Europe, Africa and Asia, and is used in various culinary, pharmaceutical and cosmetics industries [94]. There are three species in the *Rosmarinus* genus, *R. officinalis*, *R. eryocalix* and *R. tomentosus*, but *R. officinalis* is the most widely distributed and important for its valuable EO, which can be extracted in amounts ranging from 0.9 to 2.5%, depending on many factors [93,95,96]. Rosemary EO is appreciated in cosmetics for its strong camphorous aroma, and in medicine for its content in biological compounds of high value [87,97].

The compounds generally found to be dominant in *R. officinalis* EO are 1,8-cineol, camphor, α-pinene, borneol, p-cymene and verbenone, as reported in Hernández et al. [96]. The proportions of these, however, vary considerably among chemotypes [98,99]. *R. officinalis* is one of the Lamiaceae species that has received considerable attention in plant-based bioherbicide research. Many recent studies have investigated the phytotoxicity of its extracts to weeds, and in this respect significant results were obtained on a number of important weed species, such as *A. retroflexus* L., *Bromus tectorum* L., *Cynodon dactylon* L., *Digitaria sanguinalis* L. and *L. perenne* [5,8,99]. All the studies reported a concentration-dependent effect: some even found that at very low concentrations, such as 100 or 200 μL/L, the extracts had a stimulatory effect rather than a phytotoxic one [8]. Nevertheless, for some species, significant decreases in germination were found at concentrations as low as 400 μL/L [8,99]. More resistant species (mostly monocots), however, were only sensitive to higher tested doses [5,7,8,99]. A range of concentrations were therefore always tested. As EOs are very susceptible to environmental conditions, recent studies are now investigating innovative formulations to support their practical implementation as bioherbicides. For instance, nanoformulation and encapsulation in starch were successfully tested as germination and early growth inhibitors [100].

### 4.3. Thyme

The genus *Thymus* L. from the Lamiaceae family consists of over 200 species of herbaceous perennials and small shrubs [101,102]. Many of these species are widespread in the world, but the center of the genus is considered to be in the Mediterranean region [101,103]. *Thymus* is one of the most studied genera for bioactive activity due to its wide use in folk medicine [102]. This bioactive activity can be linked to its high phenolic monoterpene content (e.g., carvacrol and thymol are the major compounds in the species EO), in addition to α-terpinene, α-cymene and borneol [6,92,101]. One of the emerging bioactivity investigations of *Thymus* spp. EO is in weed control research. Similar to oregano, low doses of EO from thyme were reported to have potent phytotoxic effects on a number of problematic weed species. For instance, Hanana et al. [92] tested the EO from *Thymus capitatus* against *S. arvensis*, *P. paradoxa* and *L. rigidum*, and germination inhibition was significant at concentrations as low as 0.25 µL/mL. A recent study by Sarić-Krsmanović et al. [104] also tested the bioherbicidal effect of *T. vulgaris* EO, which is dominated by carvacrol (17.0%), thymol (11.6%) and p-cymene (11.6%), against Abutilon theophrasti Medik. Complete germination inhibition was obtained at a concentration of 1% EO. After applying *Thymus* spp. EO, other weed species such as *A. retroflexus*, *Avena fatua* L., *Datura stramonium* L., *Lepidium sativum* L. and *Agrostemma githago* L. were also significantly affected, either at germination or at the seedling growth stage [6,105]. These findings indicate that different species of *Thymus* (*T. fallax* Fisch. et Mey, *T. vulgaris* L., *T. capitatus* (L.) Hoffmanns. & Link, *T. daenensis* Celak.), even if somewhat varied in chemical composition, have great potential as bioherbicides. Kashkooli and Saharkhiz [6] tested different ecotypes of the same species (*T. daenensis* Celak.), and despite the important chemical variations in the EOs, no significant differences were observed in their effect on weeds.

### 4.4. Mint

*Mentha* L. is another important genus of the Lamiaceae family that is widely distributed and cultivated in most parts of the world thanks to its adaptation to diverse environments. About 42 species and 15 hybrids fall under this genus: they are commonly characterized by odorous secondary metabolites that make its EO famous [106,107]. In fact, most *Mentha* species are industrially cultivated for EO production. Members of the genus *Mentha* show a great variability in chemical composition, both intra- and inter-species, resulting in different chemotypes. Nevertheless, most of the species are either C3-oxygenated p-menthane types (e.g., pulegone, menthone, menthol) or C6-oxygenated p-menthane (e.g., carvone) types [107,108]. The EOs of species from both types figure in bioherbicide research. For instance, the EOs of *M. dumetorum* Schult, *Mentha × piperita* L. cv. Mitcham (Peppermint), *M. pulegium* L. and *M. spicata* L. were successfully tested on different weed species as germination and growth inhibitors. Different studies tested them on a variety of weed species with varying results. Onaran et al. [105] found that *M. dumetorum* EO suppressed the germination of *A. theophrasti* better than other tested EOs such as *O. vulgare* and *T. fallax*. Another test used the EO from a specific Peppermint cultivar (*Mentha × piperita* L. cv. Mitcham) with 35% menthol on tomato (*Lycopersicon esculentum* Mill.) and radish (*Raphanus sativus* L.), in addition to three weeds: *Convolvulus arvensis* L., *P. oleracea* and *Echinochloa colonum* L. The EO caused varying degrees of inhibition, depending on the species; tomato was particularly sensitive, and germination was completely suppressed at 900µL/L, whereas field bindweed and purslane were still able to germinate even at 1500µL/L [109]. Similarly, a study by Argyropoulos et al. [110] assessed the effects of a *Mentha* species EO (*M. spicata*) on two horticultural crops (cotton and tomato), besides weeds (*A. retroflexus*, *E. crus-galli*, *Oryza sativa* L., *P. oleracea* and *Setaria verticillata*). The EO containing 82% trans piperitone oxide severely inhibited all the tested species, but with a greater effect on cotton. The EOs of *Mentha*, like that of the other species discussed above, mainly have dose- and species-dependent effects. The reason for this selectivity and the mechanisms involved are still unclear.

### 4.5. Other Species

With fewer occurrences in the literature than those detailed above, other species in the Lamiaceae family were used to produce bioherbicidal extracts, mainly EOs. Among these number the *Salvia*, *Satureja*, *Nepeta* and *Lavandula* species [8,79,111,112]. Findings reveal differences among tested species and in resulting toxicity levels, but all conclude that the tested monoterpene-rich EOs are promising.

## 5. Conclusions

In summary, many Lamiaceae species are valuable for the bioactive compounds they contain. They are widely distributed throughout the Mediterranean area and are currently cultivated in most parts of the world. Many yield significant amounts of EOs (up to 8%) rich in terpenes, which are considered important in allelopathic interactions, and may thus have potential as bioherbicides. To this end, a considerable amount of research is still needed on technical aspects, such as exploring the mechanism of action, understanding selectivity, investigating side effects on beneficial plants, and exploring innovative formulations for effective application. Furthermore, studies are required to assess cost-benefits and define the target value for the class crops, as well as the environmental impact of production.

## Figures and Tables

**Table 1:** Limitations to current direct weed control methods in organic farming.

Direct Method	Limitations	Reference
Mechanical weeding (Tillage/ Cultivation)	Weather and soil moisture conditions Excessive soil disturbance Difficult to control perennial weeds Damages to crops root system No/reduced tillage systems Difficult to control within rows Stimulation of latent weed seed germination Most energy-consuming task (Fossil fuel)	[27,28,30]
Flame weeding	Possible damage to the crop Effectiveness depends on weed tolerance to heat and weather conditions High machine cost initially	[27,30]
Manual weeding	Large surfaces High cost	[27]
Bioherbicides	Limited products available (only 13 registered products for organic farming; only one is based on plant extract) Nonselective products Too expensive considering the necessary rates Marginal efficacy	[2,27,32]

**Table 2:** Examples of frequently tested families for bioherbicidal activity.

Species	Family	Bio Herbicidal Effect	Reference
*Xanthium strumarium* L.	Asteraceae	Significant inhibition of germination and growth of the noxious weed *Bidens pilosa* L.	[35]
*Thymus fontanesii* Boiss. et Reut. *Satureja calamintha* subsp. *nepeta* Briq.	Lamiaceae	Wide herbicidal effect on seed germination and 3–4 leaf stage of *Sinapis arvensis* L, *Avena fatua* L., *Sonchus oleraceus* L., *Xanthium strumarium* L., *Cyperus rotundus* L.	[36]
*Ulex europaeus* L. *Cytisus scoparius* L.	Fabaceae	Pure volatile organic compounds extracted caused irreversible phytotoxicity for *Digitaria sanguinalis* L.	[37]
*Trachyspermum copticum* L.	Apiaceae	Germination and shoot/root length of *Zea mays* L. and *Lepidium sativum* L. significantly reduced by all concentrations of EO and methanol extract.	[38]
*Eucalyptus citriodora* Hook	Myrtaceae	*Parthenium hysterophorus* L.: Germination completely inhibited. Chlorophyll content and respiratory activity decreased for 4-week-old plants.	[39]

**Table 3:** Examples of monoterpenes and their phytotoxic effect.

Monoterpene	Representative Structure	Chemical Function	Containing Plant Species	Germination Inhibition	Reference
α-pinene	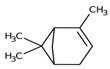	Monoene	*Eucalyptus grandis* W. Hill ex Maiden *Rosmarinus officinalis* L.	*Amaranthus hybridus* L. * Portulaca oleracea L. * Pisum sativum L. *Cicer arietinum* L.	[47] [48]
Limonene	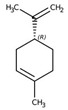	Monoene	*Citrus limon* (L.) Burm. f. *Apium graveolens* L.	*Amaranthus viridis* L. *	[49]
1,8-cineole	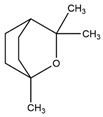	Ether	*Eucalyptus* spp. *R. officinalis* L.	*Ageratum conyzoides* L. *	[50]
α-phellandrene	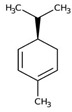	Diene	*Ligusticum marginatum* C.B. Clarke	*Raphanus sativus* L. *	[44]
Linalool	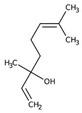	Alcohol	*Mentha* spp. *Lavandula hybrida* L.	*Echinochloa crus-galli* L. ** at highest concentration	[51]
Camphor	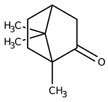	Ketone	*Lavandula abrialis* L. *R. officinalis* L.	*Amaranthus retroflexus* L. ** L. multiflorum L.** at low concentation	[14]
Pulegone	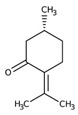	Ketone	*Mentha piperita* L. *Calamintha arkansana* (Nutt.) Shinners	*R. sativus* L. ** at low concentation	[44]
Menthol	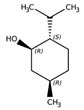	Alcohol	*Helianthus annuus* L. *Mentha* spp.	*A. retroflexus* L. ** Lolium multiflorum L. ** Lactuca sativa L.*	[14] [49]
Citronellol	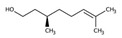	Alcohol	*Rosa* spp. *Eucalyptus* spp.	*Chenopodium album* L. * A. retroflexus L. * E. crus-galli L. ** at highest concentration	[18] [52]
Borneol	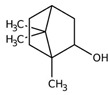	Alcohol	*Salvia officinalis* L. *R. officinalis* L.	*Lepidium sativum* L. *R. sativus* L. *	[53]
Carvacrol	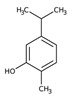	Alcohol	*Origanum vulgare* L. *Thymus capitatus* L.	*L. perenne* L. ** *A. retroflexus* L. **	[31]

* Significant effect; ** Total inhibition; *N*: no significant effect.
